# Unplanned 30-day readmissions, comorbidity and impact on one-year mortality following incident heart failure hospitalisation in Western Australia, 2001–2015

**DOI:** 10.1186/s12872-022-03020-x

**Published:** 2023-01-16

**Authors:** Courtney Weber, Joseph Hung, Siobhan Hickling, Ian Li, Kevin Murray, Tom Briffa

**Affiliations:** 1 School of Population and Global Health, University of Western Australia, Crawley, WA Australia; 2 Medical School, University of Western Australia, Crawley, WA Australia

**Keywords:** Heart failure, Hospitalisation, Readmission, Comorbidity, Mortality, Risk predictors

## Abstract

**Background:**

Readmissions within 30 days after heart failure (HF) hospitalisation is considered an important healthcare quality metric, but their impact on medium-term mortality is unclear within an Australian setting. We determined the frequency, risk predictors and relative mortality risk of 30-day unplanned readmission in patients following an incident HF hospitalisation.

**Methods:**

From the Western Australian Hospitalisation Morbidity Data Collection we identified patients aged 25–94 years with an incident (first-ever) HF hospitalisation as a principal diagnosis between 2001 and 2015, and who survived to 30-days post discharge. Unplanned 30-day readmissions were categorised by principal diagnosis. Logistic and Cox regression analysis determined the independent predictors of unplanned readmissions in 30-day survivors and the multivariable-adjusted hazard ratio (HR) of readmission on mortality within the subsequent year.

**Results:**

The cohort comprised 18,241 patients, mean age 74.3 ± 13.6 (SD) years, 53.5% males, and one-third had a modified Charlson Comorbidity Index score of ≥ 3. Among 30-day survivors, 15.5% experienced one or more unplanned 30-day readmission, of which 53.9% were due to cardiovascular causes; predominantly HF (31.4%). The unadjusted 1-year mortality was 15.9%, and the adjusted mortality HR in patients with 1 and ≥ 2 cardiovascular or non-cardiovascular readmissions (versus none) was 1.96 (95% confidence interval (CI) 1.80–2.14) and 3.04 (95% CI, 2.51–3.68) respectively. Coexistent comorbidities, including ischaemic heart disease/myocardial infarction, peripheral arterial disease, pneumonia, chronic kidney disease, and anaemia, were independent predictors of both 30-day unplanned readmission and 1-year mortality.

**Conclusion:**

Unplanned 30-day readmissions and medium-term mortality remain high among patients who survived to 30 days after incident HF hospitalisation. Any cardiovascular or non-cardiovascular readmission was associated with a two to three-fold higher adjusted HR for death over the following year, and various coexistent comorbidities were important associates of readmission and mortality risk. Our findings support the need to optimize multidisciplinary HF and multimorbidity management to potentially reduce repeat hospitalisation and improve survival.

**Supplementary Information:**

The online version contains supplementary material available at 10.1186/s12872-022-03020-x.

## Introduction

Heart failure (HF) is a global public health problem, with considerable societal and economic burden associated with high hospitalisation rates and mortality [1–3]. Concomitantly, early readmission rates, which can reflect insufficient quality of in-hospital and outpatient care are associated with adverse outcomes [1, 4]. A study conducted between 2010 and 2015 across Australia and New Zealand found that within 30 days of HF hospitalisation, one in 10 patients died, and almost a quarter of those surviving experienced at least one unplanned readmission [5]. Similarly high 30-day readmission and mortality rates were reported in Medicare beneficiaries aged 65 years and older after HF hospitalisation in the United States (US) [6]. Risk of these outcomes varied among hospitals, suggesting disparities in HF care [5, 6]. Therefore, health policy agencies widely regard 30-day all-cause readmissions as a measure of quality care in HF patients [7].

The US Hospital Readmission Reduction Program was introduced in 2012 to reduce 30-day readmissions in HF patients and to standardise care, through financial penalties for hospitals with disproportionate risk-adjusted readmission rates [8]. This program resulted in smaller than anticipated reductions in risk-standardised readmission rates [9]. with ongoing debate about whether 30-day readmission reduction initiatives may paradoxically worsen patient outcomes [8, 10]. Further, studies have suggested a poor correlation between 30-day readmission and mortality [9, 11]. As HF hospitalisation is associated with a high case-fatality, and since death is a competing risk with readmissions, it is crucial to perform a landmark analysis to assess the impact of 30-day readmission on subsequent medium-term mortality, which is currently unclear [9, 12].

We therefore assessed the frequency and reasons for 30-day unplanned readmissions in patients after incident (first-ever) hospitalisation in Western Australia (WA) between 2001 and 2015, and their impact on all-cause mortality over 1-year follow-up in 30-day survivors. The secondary aim was to determine predictors of 30-day unplanned readmission and/or mortality, as these factors may inform on risk stratification and interventions to improve outcomes.

## Methods

### Data sources

Statutory hospitalisation and death records for all patients with a cardiovascular-related hospitalisation between 1 Jan 1986 and 31 Dec 2016 were obtained from two routinely collected WA datasets, the Hospital Morbidity Data Collection (HMDC) and the Death Registry. These datasets are regularly linked through the use of probabilistic matching, and continuously audited for quality control [13]. The HMDC includes all private and public hospitalisation records, containing the dates of admission and discharge, the principal diagnosis field and 20 additional diagnosis fields, and 11 procedural fields. The Death Registry includes all demographics, date of death, and underlying and associated causes of death for those that occur within WA.

### Cohort

**Fig. 1:**
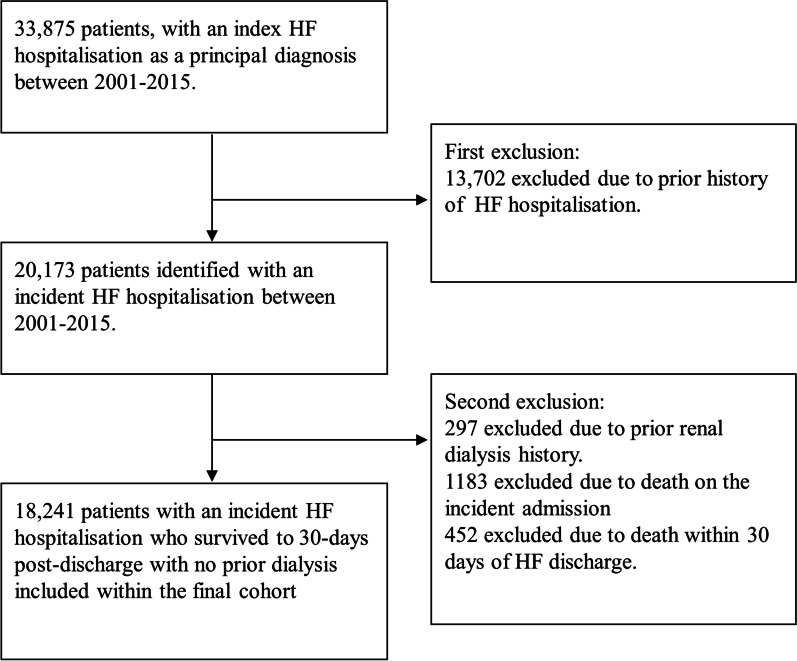
Flow diagram of cohort selection

We identified patients, aged 25–94 years, with an incident hospitalisation for HF as a principal diagnosis using the International Classification of Diseases, version 9 (ICD-9) and version 10- Australian modification (ICD-10-AM), coding definitions between 1 Jan 2001 and 31 Dec 2015 (see Additional file 1: Table S1). The coding of HF using the HMDC has previously been validated [14]. Prevalent HF cases were excluded on the basis of any HF hospitalisation record within a 10-year lookback period. Patients who died in-hospital or within 30 days of discharge were excluded as well as those on chronic renal dialysis due to their expected high readmission rates (Fig. 1).

### Covariates

Age, sex, and length of hospital stay (LOS) were collected from the incident admission. A previously constructed method, using a 15 year lookback period, identified Indigenous and Torres Strait Islander status (hereby referred to as Indigenous) [15]. A 10-year lookback period was used to identify comorbid conditions/procedures using any diagnosis field within hospitalisation records. These conditions included atrial fibrillation (AF), hypertension, ischaemic heart disease (IHD), myocardial infarction (MI), prior coronary artery bypass graft or percutaneous coronary intervention procedure (CABG/PCI), cerebrovascular disease, stroke including transient ischaemic attack (TIA) and pulmonary embolism, peripheral arterial disease (PAD), valvular heart disease, syncope, chronic obstructive pulmonary disease (COPD), chronic kidney disease (CKD), cancer, obesity, diabetes, pneumonia, anaemia, and thyroid disease (conditions identified using ICD-9 and ICD-10-AM codes shown in Additional file 1: Table S1). A modified Charlson Comorbidity Index (CCI), excluding HF, was calculated based on published coding algorithms [16].

### Outcomes

Patients surviving 30-days post-discharge were followed for 1 year or until death, whichever occurred first. The primary outcomes of interest were the frequency and reasons for 30-day unplanned readmissions, and 1-year all-cause mortality in 30-day survivors. Readmissions were counted and categorised using the principal diagnosis for each episode of care, but hospitalisation records that occurred within one day of a readmission record were considered transfers and not counted. Readmissions were stratified by elective or unplanned (emergent) status, with unplanned readmissions then categorised by principal diagnosis into disease groupings. Groups were then separated into cardiovascular or non-cardiovascular causes (coding definitions, Additional file 1: Table S2). The secondary outcomes were the temporal trends in 30-day readmission rates between 2001 and 2015, and independent predictors of 30-day unplanned readmission and all-cause mortality in 30-day survivors.

#### Statistical analysis

Continuous variables are reported as mean and standard deviation (SD) and categorical variables as number and proportion. For pairwise comparisons, *t*-tests and χ^2^ tests were used. Unplanned 30-day readmission rates were calculated and standardised to the age- and sex-distribution of incident HF patients admitted in 2015. Poisson regression analysis estimated the age-group and sex-adjusted annual rate ratio (ARR) of 30-day readmission, with 95% confidence interval (CI), between 2001 and 2015.

Multivariable logistic regression models determined covariates independently associated with 30-day unplanned readmission, presented as odds ratios (ORs) with 95% CIs. Sex, age-group (25–54, 55–64, 65–74, 75–84, 85–94 years), Indigenous status, calendar admission year, LOS, and all univariately associated (*p* < 0.05) baseline comorbidities (Table 1) with no high levels of collinearity were included in the multivariable analysis. However, results of Indigenous status are not shown to respect Indigenous data sovereignty.

**Table 1:** Characteristics of 30-day survivors of incident heart failure between 2001 and 2015, by presence of unplanned readmission

Clinical characteristics	Total	30-day readmission	
		None	Any
Count *n (%)*	18241	15421 (85.5)	2820 (15.5)
Age years, *mean (SD)*	74.3 (13.6)	74.0 (13.7)	75.5 (13.5)***
*Age group n (%)*			
25–54	1940 (10.6)	1677 (10.9)	263 (9.3)
55–64	2215 (12.1)	1906 (12.4)	309 (11.0)
65–74	3676 (20.2)	3179 (20.6)	497 (17.6)
75–84	6046 (33.2)	5052 (32.8)	994 (35.3)
85–94	4364 (23.9)	3607 (23.4)	757 (26.8)***
Men *n (%)*	9750 (53.5)	8249 (53.5)	1501 (53.2)
Indigenous status *(%)*	729 (4.0)	574 (3.7)	155 (5.5)***
LOS days *median, (IQR)*	4 (2–9)	4 (2–9)	5 (2–9)
*Comorbidities n (%)*			
Atrial fibrillation	6974 (38.2)	5833 (37.8)	1141 (40.5)**
Hypertension	10493 (57.5)	8758 (56.8)	1735 (61.5)***
Ischaemic heart disease	7422 (40.7)	6166 (40.0)	1256 (44.5)***
Myocardial infarction	2790 (15.3)	2293 (14.9)	497 (17.6)***
Prior CABG/PCI	2104 (11.5)	1735 (11.3)	369 (13.1)
Cerebrovascular disease	2186 (12.0)	1797 (11.7)	389 (13.8)**
Stroke	1947 (10.7)	1605 (10.4)	342 (12.1)*
Peripheral arterial disease	2098 (11.5)	1701 (11.0)	397 (14.1)***
Valvular disease	3582 (19.6)	2986 (19.4)	596 (21.1)*
Syncope	1233 (6.8)	995 (6.5)	238 (8.4)***
COPD	3293 (18.1)	2682 (17.4)	611 (21.7)***
Pneumonia	2933 (16.1)	2391 (15.5)	542 (19.2)***
Chronic kidney disease	3639 (20.0)	2945 (19.1)	694 (24.6)***
Cancer	5389 (29.5)	4495 (29.2)	894 (31.7)**
Diabetes	5415 (29.7)	4483 (29.1)	932 (33.1)***
Obesity	1856 (10.2)	1561 (10.1)	295 (10.5)
Anaemia	2863 (15.7)	2335 (15.1)	528 (18.7)***
Thyroid disease	620 (3.4)	491 (3.2)	129 (4.6)***
CCI score‡ *median, IQR*	2 (0–3)	2 (0–3)	2 (1–4)
*CCI score‡ n (%)*			
0	5236 (28.7)	4608 (29.9)	628 (22.3)
1–2	6526 (35.8)	5550 (37.0)	976 (34.6)
3 +	6479 (35.5)	5263 (34.1)	1216 (43.1)***

*LOS* length of hospital stay; *IQR* interquartile range; *CABG/PCI* coronary artery bypass graft/percutaneous coronary intervention; *COPD* chronic obstructive pulmonary disease; *CCI* Charlson Comorbidity Index

^‡^CCI score did not include heart failure as a comorbidity

Asterix denotes result is significant at *p*-value < 0.05 (*), *p*-value < 0.01 (**) or *p*-value < 0.001 (***)

To avoid immortal time bias, a landmark analysis at 30-days post-discharge was performed with only patients surviving to that point included in the analysis [17]. The cumulative incidence of 1-year mortality in 30-day survivors was estimated using Kaplan–Meier methods stratified by frequency of 30-day unplanned readmissions (0, 1, or 2 +). Multivariable Cox proportional hazards models, including the same covariates mentioned above, were used to determine the independent predictors of mortality over 1-year in 30-day survivors, presented as hazard ratios (HRs) and 95% CIs. Separate Cox regression models determined the mortality HR of any and number (1, 2 +) of 30-day unplanned readmission (versus no readmission), for HF-specific readmission, and for readmission from all cardiovascular or non-cardiovascular causes. Interactions tested between age-group, sex and admission-year were non-significant and therefore not included in the final Cox regression models. The proportional hazards assumption was tested by determining the interaction between the covariates and the log of time, which demonstrated no major violations. A result was considered statistically significant (*p* < 0.05) where the 95% CI around an OR/HR did not cross unity. The statistical analysis was conducted in SAS version 9.4 for Windows.

## Results

### Cohort characteristics

Among 33,875 patients, aged 25–94 years, with a first-in-period HF hospitalisation between 2001 and 2015, 13,702 (40.4%) were excluded due to a prior HF hospitalisation history. A further 1635 were excluded due to death in-hospital (*n* = 1183) or within 30 days post-discharge (*n* = 452) giving a 30-day case-fatality of 8.1%; another 297 were excluded due to chronic renal dialysis (Fig. 1). The mean age of the remaining 18,241 patients surviving 30 days after incident HF hospitalisation was 74.3 years (SD 13.6), with 53.5% male, and 4.0% Indigenous.

Table 1 shows the baseline characteristics of the study cohort, stratified by presence of any 30-day unplanned readmission. Prevalent comorbidities (≥ 15%) in the overall cohort included AF, hypertension, IHD, valvular heart disease, COPD, CKD, pneumonia, cancer, diabetes, and anaemia, and 36% had a modified CCI score ≥ 3. Females compared to males were generally older with a higher prevalence of hypertension, anaemia and thyroid disease but a lower prevalence of IHD, PAD, CKD, and diabetes (Additional file 1: Table S3).

In the cohort, any 30-day unplanned readmission occurred in 15.5% (*n* = 2820), with 13.7% (n = 2503) having one readmission only and 1.8% (*n* = 317) with 2 + readmissions. Patients with any, versus no readmission, were older, more likely identified as Indigenous but with no gender predilection, and had a higher frequency of all surveyed comorbidities, also reflected by higher CCI scores (Table 1). As expected, those patients who died within 30-day post-discharge compared to the study cohort were considerably older, had more comorbidities and a much longer initial LOS, and were more likely to experience nonfatal readmissions prior to death (44.7% versus 15.5%) (Additional file 1: Table S4).

### Unplanned 30-day readmission trends

Trends in overall age-and sex-standardised 30-day unplanned readmission rates per 1000 population between 2001 and 2015, and stratified by sex, are shown in Additional file 1: Table S5. Overall, the annual age and sex-standardised rate of 30-day unplanned readmission was 155.3 (95% CI, 149.5–161.0), and was similar between males and females (155.5 [95% CI, 147.6–163.4] and 155.0 [95% CI, 146.6–163.4] respectively). Trend analyses indicated a non-significant annual increment over the study period of 0.9% (95% CI, 0.0–1.7%) (*p* = 0.052), which was not different between sexes (Additional file 1: Table S5).

### Causes of 30-day unplanned readmissions

Among a total of 3175 readmissions, 53.9% were principally for a cardiovascular condition, with HF (31.4%) being the most common, followed by IHD (10.4%), and arrhythmias (5.1%) (Table 2). Non-cardiovascular conditions were responsible for 46.1% of all readmissions, with COPD, pneumonia, and other respiratory disease combined (9.4%) being the most common, followed by injury and poisoning/other consequences of external causes (5.0%), gastrointestinal diseases (4.7%), and then renal failure and electrolyte imbalance (3.9%) (Table 2).

**Table 2:** Causes for 30-day unplanned readmissions after hospital discharge in incident heart failure patients surviving 30-days

Cardiovascular causes	Count	Percent
	1711	53.9
Heart failure	998	31.4
Myocardial infarction	186	5.9
Other ischaemic heart disease	143	4.5
All arrhythmias	163	5.1
Cerebrovascular disease	41	1.3
Valvular disease	32	1.0
Syncope	28	0.9
Embolism and thrombosis	19	0.6
Peripheral arterial disease	11	0.4
Other cardiovascular disease	90	2.8

Non-cardiovascular causes	1464	46.1
Chronic obstructive pulmonary disease	117	3.7
Other respiratory	98	3.1
Pneumonia	81	2.6
Injury, poisoning and certain other consequences of external causes	158	5.0
Gastrointestinal disease	150	4.7
Renal failure and fluid and electrolyte abnormality	124	3.9
Diabetes	64	2.0
Disease of the musculoskeletal system and connective tissue	78	2.5
Cancer	81	2.6
Mental health	62	2.0
Diseases of the skin and subcutaneous tissue	40	1.3
Infection	47	1.5
Urinary tract infection	48	1.5
Anaemia	39	1.2
Diseases of the genitourinary system	26	0.8
Diseases of the nervous system	26	0.8
Bleeding	19	0.6
Other unspecified conditions^**†**^	206	6.5
**Total**	**3175**	**100.0**

^†^Other unspecified categories include conditions that could not be categorised into groups containing more than 0.5% of all readmissions

### Predictors of 30-day unplanned readmission

Table 3 shows the univariate and multivariable-adjusted predictors of 30-day unplanned readmission. A higher age-group, female sex, calendar admission year, LOS, and all surveyed cardiovascular and non-cardiovascular comorbidities excluding obesity were significant (positive) univariate predictors of readmission (all *p* < 0.05). After multivariable adjustment, a higher age-group, calendar admission year, and a prior history of IHD, PAD, syncope, COPD, CKD, anaemia, and thyroid disease remained independent predictors for readmission (all *p* < 0.05).

**Table 3:** Logistic regression models of 30-day unplanned readmission predictors in incident heart failure patients surviving 30-days

Baseline variable	Univariate	Multivariable
	OR (95% CI)	OR (95% CI)
Age-group (per 10 year increment above age 25–54 years)	1.09 (1.06–1.13)***	1.08 (1.04–1.12)***
Sex (female versus male)	1.16 (1.09–1.23)*	0.99 (0.91–1.08)
Length of stay	1.00 (1.00–1.01)	1.00 (0.99–1.01)
Admission year	1.01 (1.00–1.02)*	1.01 (1.00–1.02)*
Atrial fibrillation	1.12 (1.03–1.21)**	1.06 (0.97–1.15)
Hypertension	1.22 (1.12–1.32)***	1.01 (0.92–1.12)
Ischaemic heart disease	1.21 (1.11–1.31)***	1.10 (1.01–1.20)*
Myocardial infarction^‡^	1.23 (1.10–1.36)***	–
CABG/PCI	1.19 (1.05–1.34)*	1.08 (0.90–1.30)
Cerebrovascular disease	1.19 (1.04–1.25)**	1.02 (0.89–1.17)
Stroke^‡^	1.14 (0.99–1.32)	–
Peripheral arterial disease	1.32 (1.18–1.49)***	1.15 (1.01–1.30)*
Valvular disease	1.12 (1.01–1.23)*	1.08 (0.98–1.20)
Syncope	1.34 (1.15–1.55)***	1.19 (1.02–1.38)*
Chronic obstructive pulmonary disease	1.31 (1.19–1.45)***	1.23 (1.11–1.36)***
Pneumonia	1.30 (1.17–1.44)***	1.11 (1.00–1.24)
Chronic kidney disease	1.38 (1.26–1.52)***	1.19 (1.08–1.32)***
Cancer	1.13 (1.04–1.23)**	1.06 (0.97–1.16)
Diabetes	1.20 (1.11–1.31)***	1.10 (1.00–1.21)
Obesity	1.04 (0.91–1.18)	–
Anaemia	1.29 (1.16–1.43)***	1.12 (1.01–1.25)*
Thyroid disease	1.46 (1.20–1.78)***	1.37 (1.12–1.68)**
Charlson comorbidity index score^‡§^	1.03 (1.01–1.04)***	–

*OR* odds ratio; *CI* confidence interval; *CABG/PCI* coronary artery bypass graft/percutaneous coronary intervention

^‡^Not included within the multivariable analysis due to collinearity with other included variables

^§^Charlson comorbidity index score did not include heart failure as a comorbidity

Asterix denotes result is significant at *p*-value < 0.05 (*), *p*-value < 0.01 (**) or *p*-value < 0.001 (***)

### Predictors of all-cause mortality over 1-year in 30-day survivors

**Fig. 2:**
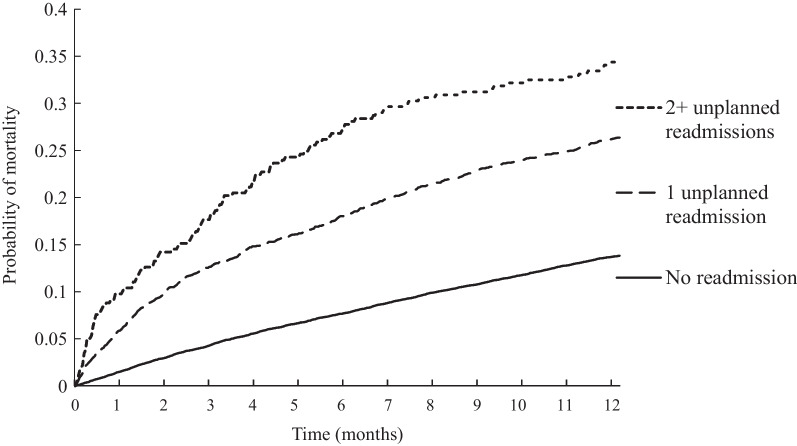
Cumulative incidence of all-cause mortality in incident heart failure patients surviving 30-days, by readmission frequency

Among 30-day survivors, 2900 (15.9%) died within 1-year. Among patients who experienced one (*n* = 2503) and 2 + (*n* = 317) unplanned 30-day readmission, the cumulative probability of all-cause death over the subsequent year was 26.4% and 34.7% respectively compared to 13.8% in patients (*n* = 15,421) without any unplanned readmission (both *p* < 0.001) (Fig. 2).

Table 4 shows univariate and multivariable-adjusted predictors of 1-year all-cause mortality in 30-day survivors. A higher age-group, LOS, and all surveyed comorbidities excluding diabetes were positive univariate predictors of mortality, while prior CABG/PCI and obesity were negative predictors (all *p* < 0.0001). After multivariable adjustment these same factors remained independent predictors of mortality except AF, valvular disease, syncope, COPD, and thyroid disease were no longer significant, and male sex became an independent positive predictor of mortality (Table 4).

**Table 4:** Cox-proportional hazards models showing 1-year all-cause mortality predictors in incident heart failure patients surviving 30-days

Variable	Univariate HR (95% CI)	Multivariable HR (95% CI)
Age (per 10 year increment above age 25–54 year)	1.67 (1.61–1.73)***	1.60 (1.54–1.67)***
Sex (female vs male)	0.96 (0.89–1.03)	0.85 (0.78–0.91)***
Length of stay	1.01 (1.01–1.01)***	1.01 (1.01–1.01)***
Admission year	1.00 (0.99–1.01)	0.99 (0.98–1.00)
Atrial fibrillation	1.20 (1.11–1.29)***	0.93 (0.86–1.01)
Hypertension	1.25 (1.16–1.34)***	0.91 (0.84–0.99)*
Ischaemic heart disease^‡^	1.07 (0.99–1.15)	–
Myocardial infarction	1.39 (1.27–1.53)***	1.31 (1.19–1.45)***
CABG/PCI	0.87 (0.77–0.98)***	0.66 (0.58–0.75)***
Cerebrovascular disease^‡^	1.70 (1.54–1.89)***	–
Stroke	1.64 (1.47–1.83)***	1.19 (1.07–1.33)***
Peripheral arterial disease	1.76 (1.60–1.94)***	1.33 (1.20–1.47)***
Valvular disease	1.18 (1.08–1.29)***	1.07 (0.98–1.17)
Syncope	1.38 (1.21–1.57)***	0.98 (0.86–1.11)
COPD	1.29 (1.18–1.41)***	1.06 (0.97–1.16)
Pneumonia	1.60 (2.46–1.74)***	1.30 (1.18–1.42)***
Chronic kidney disease	1.85 (1.71–2.01)***	1.49 (1.37–1.62)***
Cancer	1.63 (1.51–1.76)***	1.36 (1.26–1.46)***
Diabetes	1.04 (0.96–1.12)	0.99 (0.91–1.08)
Obesity	0.74 (0.65–0.85)***	0.93 (0.81–1.07)
Anaemia	1.69 (1.55–1.84)***	1.22 (1.11–1.33)***
Thyroid disease	1.17 (0.97–1.41)***	0.93 (0.77–1.13)
Charlson comorbidity index score^‡§^	1.05 (1.05–1.06)***	–
*Unplanned readmissions*		
No readmission (reference)	–	–
Any readmission	2.20 (2.03–2.39)***	2.06 (1.90–2.24)***
No readmission (reference)	–	–
1 readmission only	2.11 (1.93–2.30)***	1.96 (1.80–2.14)***
2 + readmission	3.02 (2.49–3.65)***	3.04 (2.51–3.68)***

*HR* hazard ratio; *CI* confidence interval; *CABG/PCI* coronary artery bypass graft/percutaneous coronary intervention; *COPD* chronic obstructive pulmonary disease

^‡^Not included within the multivariable analysis due to collinearity with other included variables

^§^Charlson comorbidity index score did not include heart failure as a comorbidity

Asterix denotes result is significant at *p*-value < 0.05 (*), *p*-value < 0.01 (**) or *p*-value < 0.001 (***)

Patients with any 30-day unplanned readmission (versus none) had an adjusted-HR of 2.06 (95% CI, 1.90–2.24) for 1-year mortality, and the adjusted mortality HR increased from 1.96 (95% CI, 1.80–2.14) to 3.04 (95% CI, 2.51–3.68) for 1 and 2 + unplanned readmission respectively. The adjusted mortality HR for any versus no HF readmission was 1.92 (95% CI, 1.69–2.17); for any versus no cardiovascular readmission the adjusted mortality HR was 1.71 (95% CI, 1.53–1.90), and for any versus no readmission from a non-cardiovascular cause, the mortality HR was 2.21 (95% CI, 2.00–2.45).

## Discussion

This contemporary WA population-based study demonstrated that nearly one in six experienced one or more unplanned 30-day readmissions after incident HF hospitalisation, and among 30-day survivors, a similar proportion died within 1-year follow-up. Recurrent HF was the main reason for early readmission, accounting for one-third of all events, but nearly half of all readmissions was due to non-cardiovascular conditions. Further, the frequency of any unplanned readmission was associated with a two- to threefold excess risk of all-cause death within 1 year. Importantly, prevalent cardiovascular and non-cardiovascular comorbidities independently predicted both 30-day unplanned readmission and mortality, indicating the need to optimize HF and multimorbidity management in an effort to reduce repeat hospitalisations and improve prognosis.

### Frequency and causes of readmissions

Although mortality rates have improved in hospitalised HF patients in most high-income countries due to enhanced HF treatments [2, 3, 5, 14], rates of 30-day rehospitalisation remain high despite policy intervention [5, 9, 18]. Compared to previous studies reporting all-cause readmissions, our study focused on unplanned (potentially avoidable) readmissions, and found that 15.5% of our incident hospitalised HF cohort experienced one or more unplanned readmission within 30 days of discharge. Further, this proportion did not change significantly between 20 and 2015 despite advances in HF treatments. Conversely, Labrosciano et al. [5] reported a modest decline in 30-day unplanned readmission rates from 23.2 to 21.9% in hospitalised HF patients across Australia and New Zealand between 2010 and 2015, although readmission rates varied significantly between hospitals. Their study cohort comprised a large proportion of prevalent HF cases (37%), and consequently their patients were overall older (mean age 79 years) and had a higher 30-day mortality (10.7%) compared with our incident HF cohort [5]. These differences likely explain why the overall 30-day readmission rates of our incident HF cohort was substantially lower and did not show a temporal decline [5].

Our study confirms that over half of all 30-day unplanned readmissions are for cardiovascular events, predominantly HF [18–20]. Clinical guidelines on reducing HF hospitalisations have focused on HF-specific modifiers, including fluid management, monitoring, and adherence to evidence-based pharmacological treatments [2, 3, 18]. Since our study, new HF therapies have been developed, namely the angiotensin receptor-neprilysin and sodium glucose co-transporter 2 (SGLT2) inhibitors, which have proven to be effective in reducing risk of HF hospitalisation and mortality in patients with chronic HF and reduced ejection fraction (HFrEF) [2, 3]. Importantly, these agents can also be safely initiated in-hospital following clinical stabilisation in patients with acute decompensated HF [2]. Our current data will provide a basis to assess the clinical outcome and healthcare impact of these new pharmacological agents as they become cornerstone therapies for HFrEF. However, our study also reveals that after HF hospitalisation around half of all early readmissions are primarily for non-cardiovascular conditions, such as respiratory and kidney disease, pneumonia, injury, and poisoning [18, 19, 21, 22]. Future interventions targeting reduction in 30-day readmissions after a HF hospitalisation would benefit from an increased focus on management of non-cardiovascular comorbidities by a multidisciplinary team [23].

Over one-half of our ‘real-world’ incident HF cohort were aged ≥ 75 years, with a female preponderance in this age group, and over one-third had ≥ 3 comorbidities in addition to HF. These clinical characteristics are concordant with many population-based HF cohorts from developed countries which generally contain a high proportion of elderly with multimorbidity, and with ~ 50% who have HF with preserved ejection fraction (HFpEF) [1, 21, 24]. The broad range of causes for readmission in our cohort may partly reflect their high comorbidity burden, and their resultant high vulnerability to HF and other hospitalisations [18, 20, 22]. Coexistent comorbidities such as hypertension, IHD, AF, valvular disease, COPD, pneumonia, CKD, diabetes, and anaemia are not only prevalent in our HF cohort, but these conditions are known to aggravate HF, increase the complexity of clinical management, and predispose to repeat hospitalisations and death [20, 25]. Other studies have also shown that patients with HFpEF have a similarly elevated readmission risk as those with HFrEF, although non-cardiovascular readmissions may be more common in patients with HFpEF [21, 26].

### Impact of readmission on mortality

The impact of policy initiatives aimed at reducing 30-day readmissions after HF hospitalisation has been controversial with even suggestion of an adverse impact on short-term mortality [8, 9]. We used landmark analysis in 30-day survivors to avoid an immortal time bias and discriminate the effect of prior nonfatal hospitalisation events on subsequent survival. As expected, patients who died within 30 days of incident HF compared to survivors were much older, sicker, and more prone to nonfatal hospitalisations before death, and hence their exclusion also reduces overestimating the mortality risk attributed to readmissions.

We still observed a high 1-year mortality rate (~ 16%) among 30-day survivors, and determined that one or more unplanned 30-day readmissions were associated with a two- to threefold higher adjusted mortality risk. To our knowledge only two previous studies, confined to patients enrolled in the ASCEND-HF and CHARM trials, have also found by similar landmark analysis that 30-day readmissions after HF hospitalisation was independently associated with an increased mortality risk over the subsequent 6–38 months [22, 27]. We also found that readmission specifically for HF was associated with a two-fold higher adjusted mortality risk, consistent with previous studies [28, 29]. Further, we found that a cardiovascular or non-cardiovascular readmission was associated with an equally elevated mortality risk. This finding is consistent with a previous study also found that any non-cardiovascular or cardiovascular hospitalisation in HF patients was associated with a similar mortality risk, and the risk was consistent across the spectrum of EF [27].

### Predictors of readmission and subsequent mortality

Determining clinical predictors of 30-day readmissions and mortality is important for identifying high-risk patients, and also informing on interventions aimed at improving clinical outcomes. Expectedly, advancing age was a strong independent predictor of adverse outcomes, and males had an increased mortality risk compared with females after adjusting for age and risk characteristics. We determined that there were comorbidities, including IHD/MI, PAD, CKD, and anaemia, which independently predicted both readmissions and mortality (Tables 3 and 4). These comorbidities are commonly associated with HF, can aggravate the disease while adversely impacting on clinical management and outcomes, and are thus the focus of comorbidity management in HF guidelines [2, 3]. While we found that diabetes was a univariate predictor of 30-day readmission only, there is now substantial evidence for use of SGLT2 inhibitors to reduce HF hospitalisations and all-cause death in HFrEF patients, with or without diabetes [30].

Considering that both cardiovascular and non-cardiovascular hospitalisations are associated with similar excess risk of death, this highlights the importance of optimal comorbidity management of HF patients [2, 3, 18, 25]. This is even more important in patients with HFpEF for whom there are few evidence-based treatments [2, 3], and where their high risk of adverse outcomes is chiefly driven by concomitant comorbidities [21, 27, 31]. Notably, transitional care programs for patients with HF across the spectrum of EF have demonstrated that timely follow-up by a specialised multidisciplinary team with focus on evidence-based HF care and optimal comorbid disease management can significantly improve clinical outcomes [23].

#### Limitations and strengths

Routinely collected administrative data are less comprehensive than data purposely collected for research and do not capture potential prognostic variables like haemodynamic status and left ventricular ejection fraction. However, previous studies in ‘real-world’ HF cohorts have reported findings similar to our study with respect to patient characteristics and readmission and mortality risk. The WA population represents 11% of the Australian population, yet, the clinical characteristics of our hospitalised HF cohort are similar to other representative Australian HF cohorts [4, 5, 24]. We could not assess for socioeconomic, health system, and individual treatment factors that may impact on clinical outcomes. Being an observational study, we cannot establish a true cause-and-effect relationship between readmission and mortality, although we confirm that 30-day unplanned readmission is an important surrogate marker for mortality. A significant strength includes use of whole-of-population-based data, with individual-level record linkage, allowing a 10-year lookback for important comorbidities and virtually complete follow-up for deaths.

## Conclusion

Despite significant advances in HF treatment, unplanned 30-day readmissions after incident HF hospitalisation remains frequent and an important surrogate marker of medium-term mortality and thus metric of healthcare quality. Coexistent cardiovascular and other comorbidities in hospitalised HF patients are independently associated with readmission and mortality risk, which has important clinical implications for secondary prevention interventions and optimal transitional care. Further clinical trials are required to determine the most effective multidisciplinary disease management models to reduce repeat hospitalisations and improve survival in hospitalised HF patients.

## Supplementary Information

**Additional file 1. Supplementary Table 1**. Definitions of ICD-9 and ICD-10-AM codes for identification of comorbid diseases and procedures.** Supplementary Table 2**. ICD-10-AM codes used to categorise the principal causes of 30-day unplanned readmissions in 30-day survivors.** Supplementary Table 3**. Clinical characteristics of 30-day survivors with an incident hospitalisation for heart failure between 2001-2015, stratified by sex.** Supplementary Table 4**. Clinical characteristics of patients with an incident hospitalisation for heart failure between 2001-2015, stratified by survival within 30 days post-discharge.** Supplementary Table 5**. The overall age-and sex-standardised rates and age-standardised rates by sex, per 1000 population, of 30-day unplanned readmission in patients who survived 30-days post-discharge after incident heart failure hospitalisation between 2001 and 2015.

## Data Availability

The data that support the findings of this study are available from the WA Department of Health, Data Linkage Branch but restrictions apply to the availability of these data, which were used under license for the current study. The datasets generated during and analysed during the current study are not publicly available due to restrictions placed by the WA Department of Health, Data Linkage Branch. Data are however available from the authors upon reasonable request and with permission of the WA Department of Health, Data Linkage Branch.

## Abbreviations

ASCEND: A Study of Cardiovascular Events in Diabetes Trial
AF: Atrial fibrillation
ARR: Annual rate ratio
CABG: Coronary artery bypass graft
CCI: Charlson Comorbidity Index
CHARM: Candesartan in Heart failure: Assessment of Reduction in Mortality and morbidity Trial
CI: Confidence interval
CKD: Chronic kidney disease
COPD: Chronic obstructive pulmonary disease
HF: Heart failure
HMDC: Hospital Morbidity Data Collection
HR: Hazards ratio
HFrEF: Heart failure with reduced ejection fraction
HFpEF: Heart failure with preserved ejection fraction
ICD: International Classification of Diseases
IHD: Ischaemic heart disease
LOS: Length of hospital stay
MI: Myocardial infarction
OR: Odds ratio
PAD: Peripheral arterial disease
PCI: Percutaneous coronary intervention
SD: Standard deviation
SGLT2: Sodium-glucose cotransporter-2
TIA: Transient ischaemic attack
US: United States
WA: Western Australia
