# Supplementary material for: Unplanned 30-day readmissions, comorbidity and impact on one-year mortality following incident heart failure hospitalisation in Western Australia, 2001–2015
Source: BMC Cardiovasc Disord. 2023 Jan 16;23:25. doi: 10.1186/s12872-022-03020-x (PMC9843857; doi:10.1186/s12872-022-03020-x)
Supplement: Supplementary file 1 — Additional file 1. Supplementary Table 1. Definitions of ICD-9 and ICD-10-AM codes for identification of comorbid diseases and procedures. Supplementary Table 2. ICD-10-AM codes used to categorise the principal causes of 30-day unplanned readmissions in 30-day survivors. Supplementary Table 3. Clinical characteristics of 30-day survivors with an incident hospitalisation for heart failure between 2001-2015, stratified by sex. Supplementary Table 4. Clinical characteristics of patients with an incident hospitalisation for heart failure between 2001-2015, stratified by survival within 30 days post-discharge. Supplementary Table 5. The overall age-and sex-standardised rates and age-standardised rates by sex, per 1000 population, of 30-day unplanned readmission in patients who survived 30-days post-discharge after incident heart failure hospitalisation between 2001 and 2015. [file 12872_2022_3020_MOESM1_ESM.docx]

**Supplementary Table 1.** Definitions of ICD-9 and ICD-10-AM codes for identification of comorbid diseases and procedures.

|  | **ICD-9** | **ICD-10-AM** |
| --- | --- | --- |
| Conditions |  |  |
| Heart failure | 391.8, 402.01, 402.11, 402.91, 404.1, 404.3, 425, 428, 514, 518.4, | I50, I43, I11.0, I13.0, I13.2, I42.0, I42.5, I42.6, I42.7, I42.8, I42.9, I25.5 |
| Atrial fibrillation | 427.3 | I48 |
| Hypertension | 401-405 | I10-I15 |
| Ischaemic heart disease | 410-414 | I20-I25 |
| Myocardial infarction | 410 | I21, I22 |
| Cerebrovascular disease | 430-438 | I60-I69, G45 |
| Stroke/embolism | 430-433 436 | I60-I64, I74 |
| Peripheral arterial disease | 440-449 | I70-I79 |
| Valvular heart disease | 393-398, 424 | I05-I09, I34-I39, Q22, Q23 |
| Syncope | 780.2 | R55 |
| Chronic obstructive pulmonary disease | 490-496 | J40-J47 |
| Pneumonia | 480-486 | J10-J18 |
| Chronic kidney disease | Pre-01/07/95:  250.4, 590.0, 590.2, 590.3, 590.8, 593.0, 593.1, 593.2, 593.6, 593.9,  599.7, 753.0, 753.1, 753.2, 753.4, 996.1, V42.0, V45.1, 403, 404, 580, 581, 582, 583, 585, 586, 587, 588, 589, V56, 405.01, 405.11, 405.91, 405.02, 405.12, 405.92, 593.81, 996.81  From 01/07/95:  250.4, 590.0, 590.2, 590.3, 590.8, 593.0, 593.1, 593.2, 593.6, 593.9,  599.7, 753.0, 753.1, 753.2, 753.4, 996.1, V42.0, V45.1, 403, 404, 580, 581, 582, 583, 585, 586, 587, 588, 589, V56, 405.01, 405.11, 405.91, 405.02, 405.12, 405.92, 593.81, 996.81 | E10.2, E11.2, E12.2, E13.2, E14.2, I15.0, I15.1, N39.1, N39.2, T82.4, Z94.0, Z99.2,  I12, I13, N00, N01, N02, N03, N04, N05, N06, N07, N08, N11, N12, N14, N15, N16, N18, N19,N25, N26, N27, N28, Q60, Q61, Q62, Q63, Z49 |
| Cancer | 140-230 | C00-C99, D00-D30 |
| Diabetes | 250 | E10-E14 |
| Obesity | 278.00-278.03 | E66 |
| Anaemia | 280, 281, 283, 285 | D50-D53, D55-D59, D61-D63 |
| Thyroid disease | 242, 244 | E03, E05 |
| **Procedural codes** |  |  |
| Coronary revascularisation (Percutaneous coronary intervention and coronary artery bypass grafting) | Pre 1/07/1999:  36.01, 36.02, 36.05, 36.06, 36.07 | 38497-00-38497-07, 38500-01-38500-05, 38503-00- 38503-04, 90201-00- 90201-03  Between 01/07/1999 and 01/07/2008:  35304-0, 35305-00, 35310-01, 35310-02  Between 01/07/2006 and 01/07/2008:  35335-00, 35341-00, 38309-00, 38315-00, 35338-01, 35344-00, 35344-01  Post 1/07/2008:  38303-00, 38300-00,38312-00, 38312-01, 38318-00, 38318-01  Post 1/07/2010:  38500-00, 38503-05  Post 01/07/2013:  90218-00-90218-03 |

ICD-9, International Classification of Diseases version 9; ICD-10-AM, International Classification of Diseases version 10 Australian Modification.

**Supplementary Table 2**. ICD-10-AM codes used to categorise the principal causes of 30-day unplanned readmissions in 30-day survivors.

| **Cardiovascular causes** | **ICD-10-AM** |
| --- | --- |
| Heart failure | I40-I43.1, I50, I95-I97, J81., R57 |
| Myocardial infarction | I21-I22.9 |
| All arrhythmias | I44-I49, R00 |
| Other ischaemic heart disease | I20, I24.0-I25.9, R07 |
| Cerebrovascular disease | G45, I62-I64 |
| Valvular disease | I05-I09, I34-I35.2, T82.0, T82.6 |
| Syncope | R55 |
| Embolism and thrombosis | I26.9, I74, I82.8, |
| Peripheral arterial disease | I70-I72, |
| Other cardiovascular disease | I00, I01.1, I10-I13.2, I27.0-I33.0, I51, I66, I67, I80, I83, I87.2, I89.0, T46.0, T82.1, T82.7, T82.8, Z03.5, Z45.0, Z73.8, Z74.2 |
| **Non-cardiovascular causes** |  |
| Chronic obstructive pulmonary disease | J40-J47 |
| Other respiratory | J00-J09, J20-J34,J61-J70, J84-J98 |
| Pneumonia | J10-J18 |
| Injury, poisoning and certain other consequences of external causes | S00-T45.5, T46.2-T81.8, T83.0-T88.7 |
| Gastrointestinal | K02-K22.6, K25.7, K25.9, K29.10-K29.70, K29.8-K57.22, K57.32, K59.0- K91.8 |
| Renal failure and fluid & electrolyte abnormality | E86., E87, N17.8-N19., Z49.1 |
| Diabetes | E10-E11 |
| Disease of the musculoskeletal system and connective tissue | M00-M99 |
| Cancer | C12-D47.5, Z51.1 |
| Mental health | F00-F99, R45.89 |
| Diseases of the skin and subcutaneous tissue | L00-L99 |
| Infection | A00-B59 |
| Urinary tract infection | N39.0 |
| Anaemia | D50-D64, E61.1 |
| Diseases of the genitourinary system | G72.8,N00-N13, N20-N35.9, N40-N75.1, R18 |
| Diseases of the nervous system | G00-G43, G47-G72.0, G72.9-G92. |
| Bleeding | D68.3, I84.8, K25.0, K25.4, K25.6, K26.0, K26.4, K29.0, K29.71,K57.31, K57.33, K57.92, K92.0, K92.1, K92.2 |
| Other unspecified conditions | All other codes not listed. |
|  |  |

ICD-10-AM, International Classification of Diseases version 10 Australian Modification.

**Supplementary table 3.** Clinical characteristics of 30-day survivors with an incident hospitalisation for heart failure between 2001-2015, stratified by sex.

| **Clinical Characteristics** | **Total** | **Men** | **Women** |
| --- | --- | --- | --- |
| Count *n (%)* | 18,241 | 9750 (53.5) | 8491 (46.6) |
| Any 30-day readmission | 2920 (15.5) | 1501 (15.4) | 1319 (15.5)*** |
| Age years, *mean (SD)* | 74.3 (13.6) | 72.3 (13.8) | 76.6 (13.1) |
| Age group *n (%)* |  |  |  |
| 25-54 | 1940 (10.6) | 1216 (22.5) | 724 (8.5) |
| 55-64 | 2215 (12.1) | 1411 (14.5) | 804 (9.5) |
| 65-74 | 3676 (20.2) | 2225 (22.8) | 1451 (17.1) |
| 75-84 | 6046 (33.2) | 3119 (32.0) | 2927 (34.5) |
| 85-94 | 4364 (23.9) | 1779 (18.3) | 2585 (30.4)*** |
| Indigenous status *(%)* | 729 (4.0) | 353 (3.6) | 376 (4.4)** |
| LOS days *median, (IQR)* | 4 (2-9) | 4 (2-8) | 5 (2-9) |
| **Comorbidities *n (%)*** |  |  |  |
| Atrial fibrillation | 6974 (38.2) | 3798 (39.0) | 3176 (37.4)* |
| Hypertension | 10,493 (57.5) | 5468 (56.1) | 5025 (59.2)*** |
| Ischaemic heart disease | 7422 (40.7) | 4470 (45.9) | 2952 (34.8)*** |
| Myocardial infarction | 2790 (15.3) | 1675 (17.2) | 1115 (13.1)*** |
| Prior CABG/PCI | 2104 (11.5) | 1503 (15.4) | 601 (7.1)*** |
| Cerebrovascular disease | 2186 (12.0) | 1188 (12.2) | 998 (11.8) |
| Stroke | 1947 (10.7) | 1038 (10.7) | 909 (10.7) |
| Peripheral arterial disease | 2098 (11.5) | 1304 (13.4) | 794 (9.4)*** |
| Valvular disease | 3582 (19.6) | 1915 (19.6) | 1667 (19.6) |
| Syncope | 1233 (6.8) | 614 (6.3) | 619 (7.3)** |
| COPD | 3293 (18.1) | 1704 (17.5) | 1589 (18.7)* |
| Pneumonia | 2933 (16.1) | 1635 (16.8) | 1298 (15.3)** |
| Chronic kidney disease | 3639 (20.0) | 2109 (21.6) | 1530 (18.0)*** |
| Cancer | 5389 (29.5) | 3016 (30.9) | 2373 (28.0)*** |
| Diabetes | 5415 (29.7) | 3041 (31.2) | 2374 (28.0)*** |
| Obesity | 1856 (10.2) | 958 (9.8) | 898 (10.6) |
| Anaemia | 2863 (15.7) | 1330 (13.6) | 1533 (18.1)*** |
| Thyroid disease | 620 (3.4) | 150 (1.5) | 470 (5.5)*** |
| CCI score^‡^ *median, IQR* | 2 (0-3) | 2 (0-4) | 2 (0-3) |
| CCI score^‡^ *n (%)* |  |  |  |
| 0 | 5236 (28.7) | 2762 (28.3) | 2474 (29.1) |
| 1-2 | 6526 (35.8) | 3308 (33.9) | 2799 (37.9) |
| 3+ | 6479 (35.5) | 3680 (37.8) | 2799 (33.0)*** |

Abbreviations. LOS, length of hospital stay; IQR, interquartile range; CABG/PCI, coronary artery bypass graft/percutaneous coronary intervention; COPD, chronic obstructive pulmonary disease; CCI, Charlson Comorbidity Index. ^‡^CCI score did not include heart failure as a comorbidity. Asterix indicates result is significant at p-value <0.05 (*), p-value <0.01 (**), or p-value <0.001 (***).

**Supplementary table 4.** Clinical characteristics of patients with an incident hospitalisation for heart failure between 2001-2015, stratified by survival within 30 days post-discharge.

| **Clinical Characteristics** | **Total** | **Survived 30 days** | **Died within 30 days** |
| --- | --- | --- | --- |
| Count *n (%)* | 18,693 | 18,241 (97.6) | 452 (2.4) |
| Any 30-day readmission | 3022 (16.2) | 2820 (15.5) | 202 (44.7)*** |
| Age years, *mean (SD)* | 74.4 (13.6) | 74.3 (13.6) | 80.9 (11.3) |
| Age group *n (%)* |  |  |  |
| 25-54 | 1957 (53.4) | 1940 (10.6) | 17 (3.8) |
| 55-64 | 2237 (12.0) | 2215 (12.1) | 22 (4.9) |
| 65-74 | 3744 (20.0) | 3676 (20.2) | 68 (15.0) |
| 75-84 | 6187 (33.1) | 6046 (33.2) | 141 (31.2) |
| 85-94 | 4568 (24.4) | 4364 (23.9) | 204 (45.1)*** |
| Male *(%)* | 9973 (53.4) | 9750 (53.5) | 223 (49.3) |
| Indigenous status *(%)* | 743 (4.0) | 729 (4.0) | 14 (3.1) |
| LOS days *median, (IQR)* | 5 (2-9) | 4 (2-9) | 8 (4-17)*** |
| **Comorbidities *n (%)*** |  |  |  |
| Atrial fibrillation | 7158 (38.3) | 6974 (38.2) | 184 (40.7) |
| Hypertension | 10,742 (57.5) | 10,493 (57.5) | 249 (55.1) |
| Ischaemic heart disease | 7620 (40.8) | 7422 (40.7) | 198 (43.8) |
| Myocardial infarction | 2888 (15.5) | 2790 (15.3) | 98 (21.7)*** |
| Prior CABG/PCI | 2137 (11.4) | 2104 (11.5) | 33 (7.3)* |
| Cerebrovascular disease | 2269 (12.1) | 2186 (12.0) | 83 (18.4)*** |
| Stroke | 2023 (10.8) | 1947 (10.7) | 76 (16.8)*** |
| Peripheral arterial disease | 2166 (11.6) | 2098 (11.5) | 68 (15.1)* |
| Valvular disease | 3676 (19.7) | 3582 (19.6) | 94 (20.8) |
| Syncope | 1266 (6.8) | 1233 (6.8) | 33 (7.3) |
| COPD | 3392 (18.2) | 3293 (18.1) | 99 (21.9)* |
| Pneumonia | 3041 (16.3) | 2933 (16.1) | 108 (23.9)*** |
| Chronic kidney disease | 3766 (20.2) | 3639 (20.0) | 127 (28.1)*** |
| Cancer | 5561 (29.8) | 5389 (29.5) | 172 (38.1)*** |
| Diabetes | 5543 (29.7) | 5415 (29.7) | 128 (28.3) |
| Obesity | 1885 (10.1) | 1856 (10.2) | 29 (6.4)** |
| Anaemia | 643 (3.4) | 2863 (15.7) | 88 (19.5)* |
| Thyroid disease | 643 (3.4) | 620 (3.4) | 23 (5.1) |
| CCI score^‡^ *median, IQR* | 2 (0-3) | 2 (0-3) | 3 (1-5) |
| CCI score^‡^ *n (%)* |  |  |  |
| 0 | 5286 (28.3) | 5236 (28.7) | 50 (11.1) |
| 1-2 | 6688 (35.8) | 6526 (35.8) | 162 (35.8) |
| 3+ | 6719 (35.9) | 6479 (35.5) | 240 (53.1)*** |

Abbreviations. LOS, length of hospital stay; IQR, interquartile range; , ischaemic heart disease; CABG/PCI, coronary artery bypass graft/percutaneous coronary intervention; COPD, chronic obstructive pulmonary disease; CCI, Charlson Comorbidity Index. ^‡^CCI score did not include heart failure as a comorbidity. Asterix indicates result is significant at p-value <0.05 (*), p-value <0.01 (**), or p-value <0.001 (***).

**Supplementary Table 5.** The overall age-and sex-standardised rates and age-standardised rates by sex, per 1000 population, of 30-day unplanned readmission in patients who survived 30-days post-discharge after incident heart failure hospitalisation between 2001 and 2015.

|  | **2001** | **2002** | **2003** | **2004** | **2005** | **2006** | **2007** | **2008** | **2009** | **2010** | **2011** | **2012** | **2013** | **2014** | **2015** | **Annual rate ratio (%)** |
| --- | --- | --- | --- | --- | --- | --- | --- | --- | --- | --- | --- | --- | --- | --- | --- | --- |
| **Overall** | 144.7 | 164.5 | 139.8 | 137.9 | 146.0 | 164.0 | 136.2 | 151.3 | 165.4 | 168.1 | 163.7 | 156.3 | 149.4 | 160.6 | 166.7 | 1.009 (1.000, 1.017) |
| **Male** | 150.4 | 170.5 | 168.6 | 134.5 | 156.2 | 154.9 | 130.2 | 124.5 | 183.3 | 160.2 | 168.4 | 159.3 | 152.9 | 165.8 | 173.5 | 1.006 (0.995, 1.018) |
| **Female** | 144.9 | 159.0 | 109.9 | 142.7 | 137.8 | 175.6 | 138.6 | 178.7 | 149.5 | 176.0 | 156.9 | 148.5 | 146.8 | 161.6 | 157.6 | 1.010 (0.998, 1.023) |
